# Thermodynamically stable [4 + 2] cycloadducts of lanthanum-encapsulated endohedral metallofullerenes

**DOI:** 10.3762/bjoc.10.65

**Published:** 2014-03-25

**Authors:** Yuta Takano, Yuki Nagashima, M Ángeles Herranz, Nazario Martín, Takeshi Akasaka

**Affiliations:** 1 Institute for Integrated Cell-Material Sciences (WPI-iCeMS), Kyoto University, Sakyo-ku, Kyoto 606-8501, Japan; 2 Life Science Center of Tsukuba Advanced Research Alliance, University of Tsukuba, Tsukuba, Ibaraki 305-8577, Japan; 3 Departamento de Química Orgánica I, Facultad de Química, Universidad Complutense, E-28040 Madrid, Spain; 4 IMDEA–Nanoscience, Campus de Cantoblanco, Madrid E-28049, Spain; 5 Foundation for Advancement of International Science, Tsukuba, Ibaraki 305-0821, Japan; 6 State Key Laboratory of Materials Processing and Die & Mould Technology, School of Materials Science and Technology, Huazhong University of Science and Technology, Wuhan 430074, China and Department of Chemistry, Tokyo Gakugei University, Tokyo 184-8501, Japan

**Keywords:** carbon nanomaterials, dynamic NMR, endofullerenes, La_2_@C_80_, La@C_82_, sultine

## Abstract

The [4 + 2] cycloaddition of *o*-quinodimethanes, generated in situ from the sultine 4,5-benzo-3,6-dihydro-1,2-oxathiin 2-oxide and its derivative, to La metal-encapsulated fullerenes, La_2_@C_80_ or La@C_82_, afforded the novel derivatives of endohedral metallofullerenes (**3a**,**b**, **4a**,**b** and **5b**). Molecular structures of the resulting compounds were elucidated using spectroscopic methods such as MALDI–TOF mass, optical absorption, and NMR spectroscopy. The [4 + 2] adducts of La_2_@C_80_ (**3a**,**b**, and **4a**,**b**) and La@C_82_ (**5b**), respectively, retain diamagnetic and paramagnetic properties, as confirmed by EPR spectroscopy. Dynamic NMR measurements of **4a** at various temperatures demonstrated the boat-to-boat inversions of the addend. In addition, **5b** revealed remarkable thermal stability in comparison with the reported [4 + 2] cycloadduct of pentamethylcyclopentadiene and La@C_82_ (**6**). These findings demonstrate the utility of sultines to afford thermodynamically stable endohedral metallofullerene derivatives for the use in material science.

## Introduction

Endohedral metallofullerenes (EMFs) are a family of nanocarbons, which encapsulated one or more metal atoms inside a hollow carbon cage [1–4]. The encapsulation results in the electron transfer from metal atoms to the fullerene cage, which leads to unique electronic, magnetic, and chemical properties for EMFs that cannot be expected for empty fullerenes. Due to the numerous electronic properties EMFs are anticipated as promising materials in various fields such as chemistry, biology, and material science.

Among various kinds of EMFs, those encapsulating La atoms are especially attractive molecules because of their electronic and magnetic characteristics. As a result of the three electron transfer per La atom to the fullerene cage, the fullerenes simultaneously possess a low ionizing potential and a high electron affinity [1–2]. For mono-La endohedral fullerenes such as La@C_82_, the electron transfer results in paramagnetism of the fullerene cage [5]. The di-La endohedral fullerenes such as La_2_@C_80_ show diamagnetism [6]. This feature leads to remarkable differences in chemical and electronic properties between these two classes of EMFs.

Chemical functionalization of fullerenes enhances molecular properties and possible applications of fullerenes [1,4]. The [4 + 2] cycloaddition reaction is a useful chemical modification method because it enables to introduce a variety of addends and/or the combination of different functionalities on the fullerene [7]. Regarding the [4 + 2] cycloaddition of EMFs, however, no report is available for endohedral di-metallofullerenes, with the exception of azafullerene [8]. A limited number of reports describe other EMFs [4,9–10]. Moreover, the only precedent of [4 + 2] cycloadducts of the fullerenes which have an open-shell electronic structure of the cage, e.g., La@C_82_, are thermodynamically unstable and show retro-cycloaddition reactions [10–11]. In addition, boat-to-boat inversion of the addend of the cycloadducts of EMFs has not been well-studied to date; the investigation of this interconversion serves to demonstrate the existence of a dynamic process in the molecules and is regarded as one index of the bonding energy of the addition position of the fullerene and the addend.

Among various precursors to afford [4 + 2] cycloadducts of fullerenes, the sultine 4,5-benzo-3,6-dihydro-1,2-oxathiin 2-oxide and its derivatives are useful to afford thermodynamically stable compounds because thermolysis of sultine affords highly reactive *o*-quinodimethanes by extrusion of sulfur dioxide without production of any organic or inorganic byproduct [12–13].

Here, we present the first chemical derivatization of La_2_@C_80_ and La@C_82_ by [4 + 2] cycloaddition using sultines **1a**,**b**. The resulting products **3a**,**b**, **4a**,**b** and **5b** were characterized and their thermodynamic properties were investigated.

## Results and Discussion

### Synthesis and characterization of La_2_@C_80_ cycloadducts

**Scheme 1:**
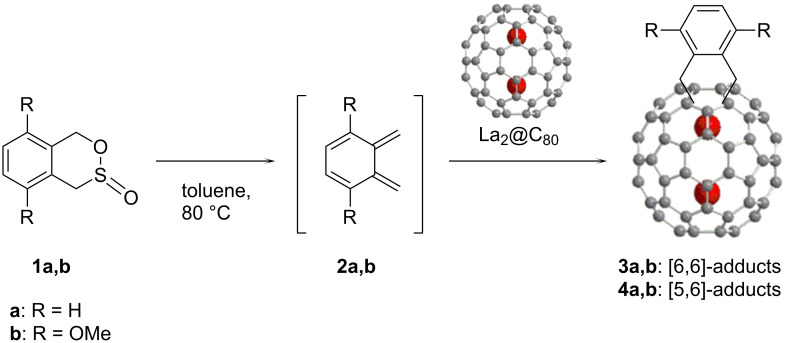
Synthesis of [4 + 2] adducts of La_2_@C_80_.

**Figure 1:**
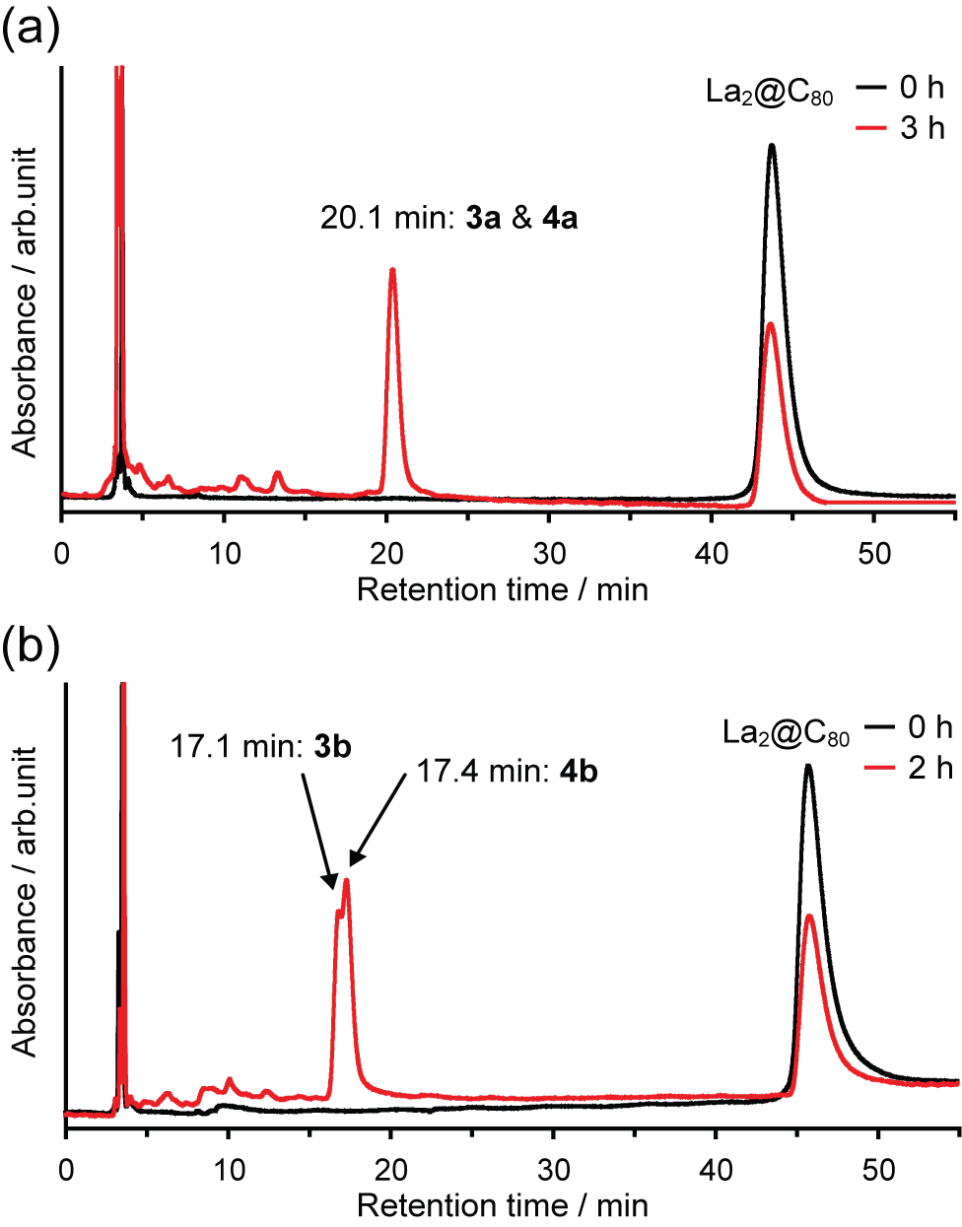
HPLC profiles of the reaction solutions (black) before and (red) after the reaction of La_2_@C_80_ and (a) **1a** and (b) **1b**, respectively. Conditions: column, Buckyprep (Ø 4.6 mm × 250 mm); eluent, toluene; flow rate, 1.0 mL/min; wavelength: 330 nm; rt.

**Figure 2:**
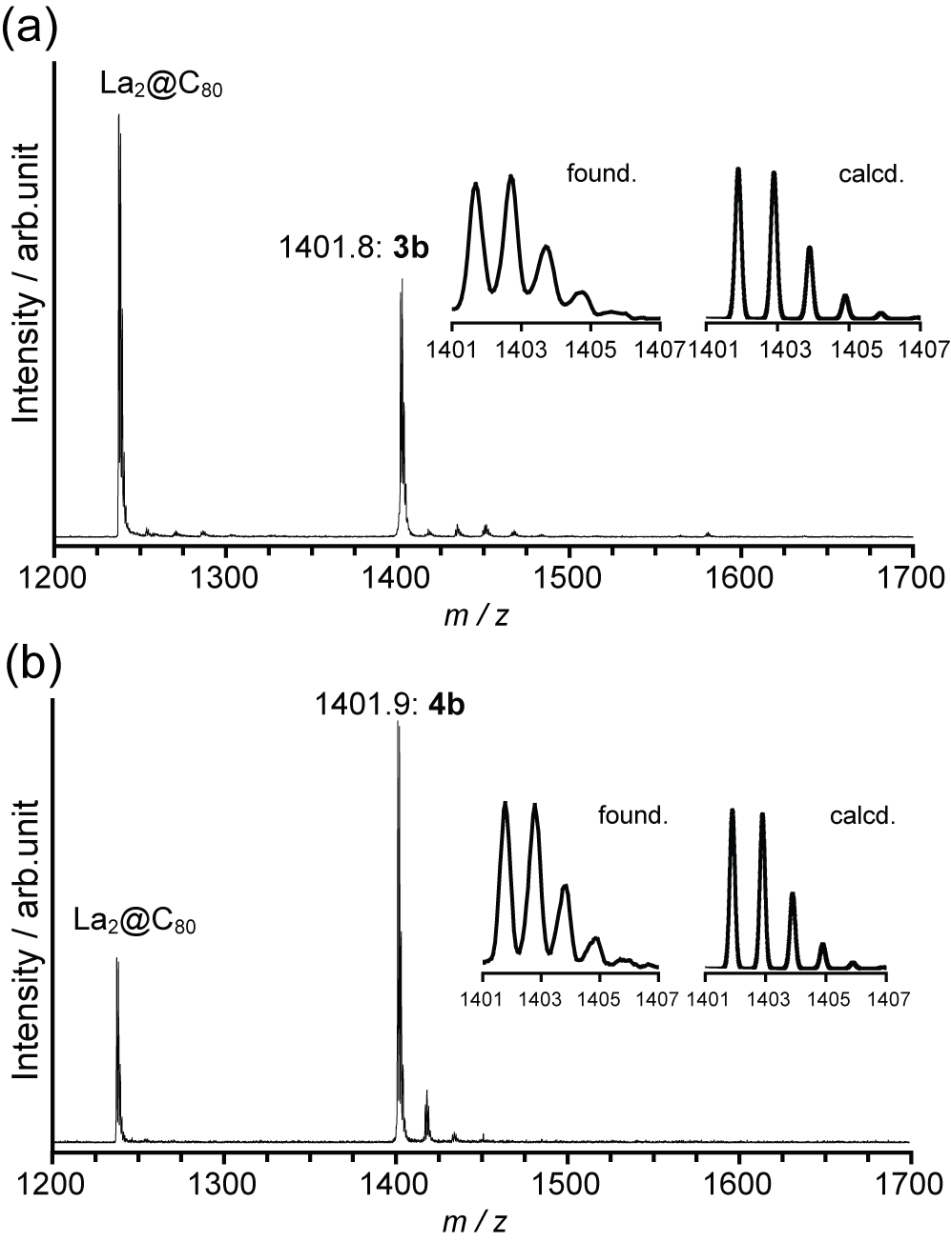
MALDI–TOF mass (negative mode) spectra of (a) **3b** and (b) **4b**, using 1,1,4,4-tetraphenyl-1,3-butadiene as matrix.

*o*-Quinodimethanes **2a** and **2b** were generated in situ by thermolysis of the corresponding sultines **1a** and **1b** in toluene at 80 °C (Scheme 1). The highly reactive intermediates are trapped efficiently by La_2_@C_80_, which acts as a dienophile to form the cycloadducts (**3a**,**b** and **4a**,**b**). The reactions were traced using HPLC analyses (Figure 1), and formation of the resulting [4 + 2] adducts was confirmed by matrix-assisted laser desorption ionization (MALDI) TOF mass spectrometry (Figure 2), which shows the molecular ion peaks for the corresponding compounds. Isolation of **3b** and **4b** were achieved by one-step HPLC separation using a Buckyprep column, although this purification method was not applicable for **3a** and **4a** (vide infra).

Because only two types of C=C bonds are available in La_2_@C_80_, which has *I_h_* symmetry, only two site-isomers namely [6,6]- and [5,6]-isomers, are allowed to be formed by cycloaddition reactions. (Please note that “site-isomer” refers to an isomer of the adducts which has the same fullerene and addend but different addition position – a classification proposed recently for fullerene’s chemistry by Martin et al. [14]). Therefore, **3b** and **4b** are concluded to be the site-isomers which were afforded by the reaction as a result of using highly reactive *o*-quinodimethane.

**Figure 3:**
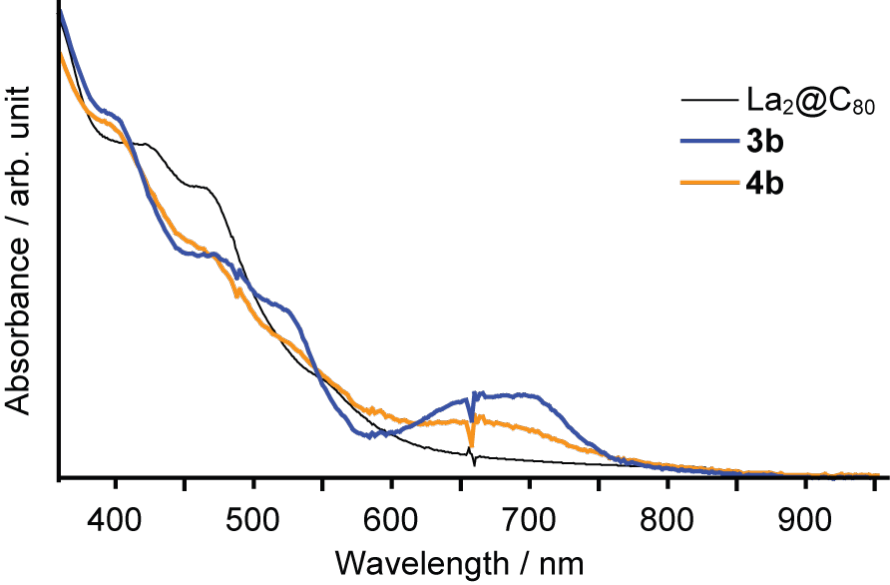
UV–vis/near-IR absorption spectra of **3b** and **4b** recorded by the diode array detector of the HPLC apparatus.

The UV–vis spectra of **3b** and **4b** partially provide information related to their molecular structures. The spectra were firstly recorded using a diode-array detector of the HPLC apparatus (Figure 3). The spectrum of **3b** shows the specific absorption band around 700 nm, which strongly suggests the electric nature of a [6,6]-closed adduct of the La_2_@C_80_ derivatives [15], because the absorption spectra of fullerenes and their derivatives are mainly attributable to π–π* transitions, which reflects the distinctive fingerprints of the π-electron system topology of the fullerene cage. Similarly, the characteristic spectrum of **4b** suggests its [5,6]-closed structure.

**Figure 4:**
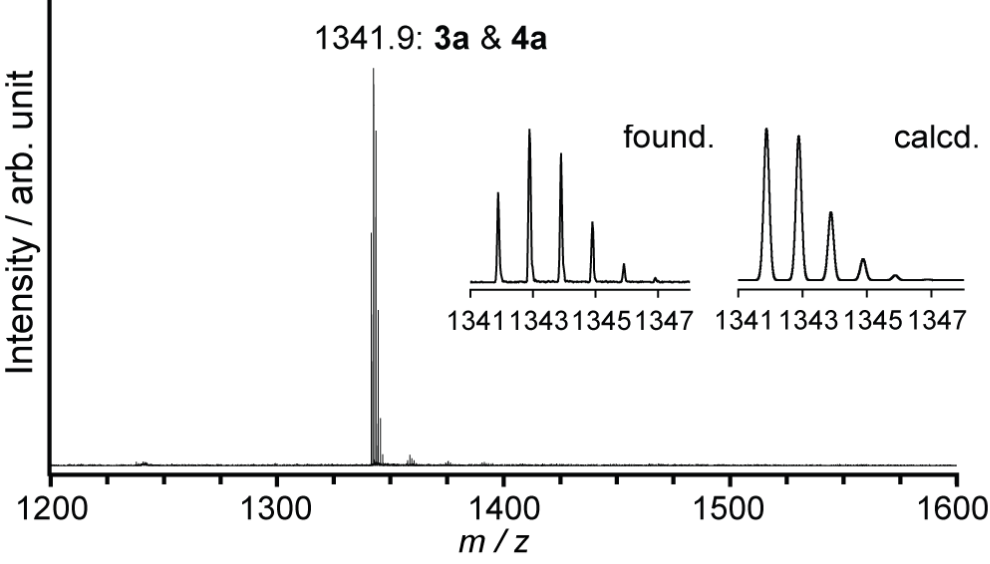
MALDI–TOF mass spectrum (negative mode) of the reaction mixture from La_2_@C_80_ and **1a**, using 1,1,4,4-tetraphenyl-1,3-butadiene as matrix.

**Figure 5:**
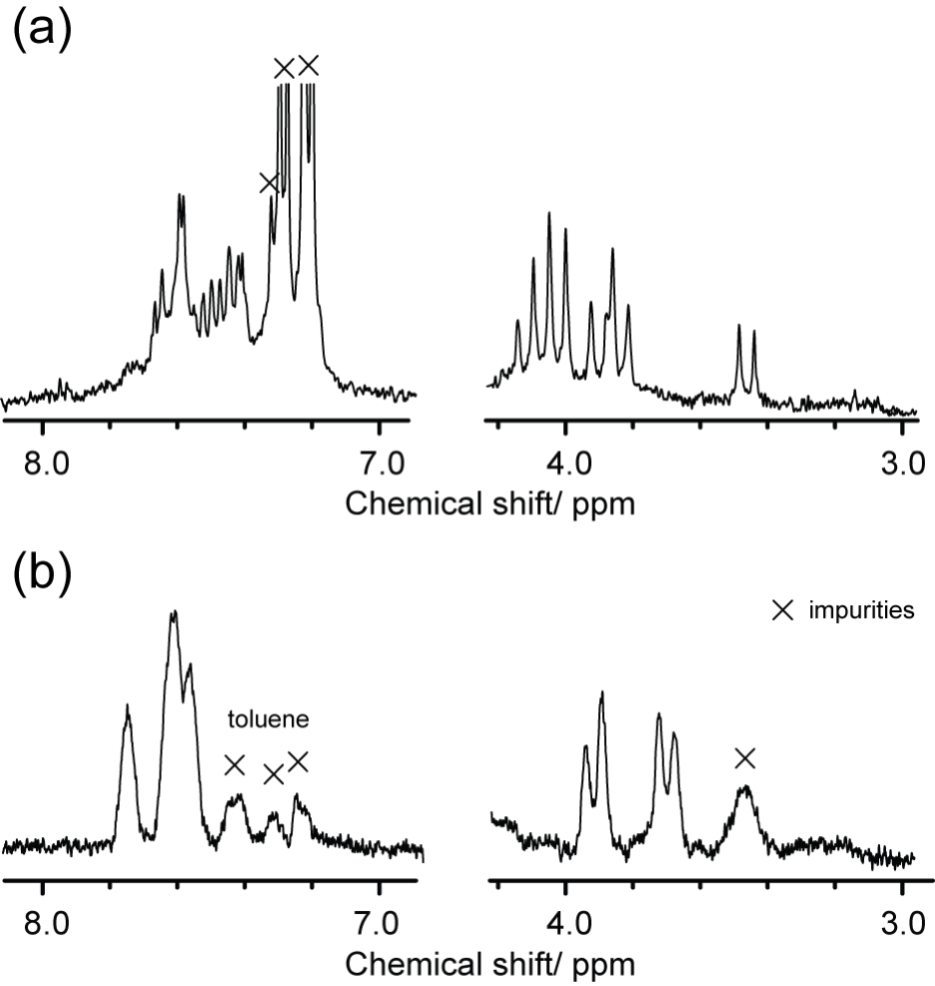
^1^H NMR spectra of (a) the mixture of **3a** and **4a** in C_2_D_2_Cl_4_ at 248 K, and (b) isolated **4a** at 230 K, recorded in 300 MHz.

A different approach was taken to purify **3a** and **4a** because of the similar retention time of these compounds in HPLC. The mixture of **3a** and **4a** was first separated from the unreacted starting materials and byproducts through one-step HPLC separation. The MALDI–TOF mass spectra of **3a** and **4a** showed single peaks attributed to the molecular ion peak of the target molecule, La_2_@C_80_C_2_H_4_C_6_H_4_, at 1342 *m*/*z* (Figure 4). The existence of both [6,6]- and [5,6]-isomers in the mixture was indicated by the ^1^H NMR spectrum recorded at 248 K (Figure 5b), which cannot be expected from a single regioisomer. The [6,6]-adduct should show only two sets of AB quartets. The [5,6]-adduct should demonstrate one or two AB quartets based on its *C_s_* molecular symmetry. Consequently, the spectrum containing more than two AB quartets indicates the existence of the both site-isomers.

**Figure 6:**
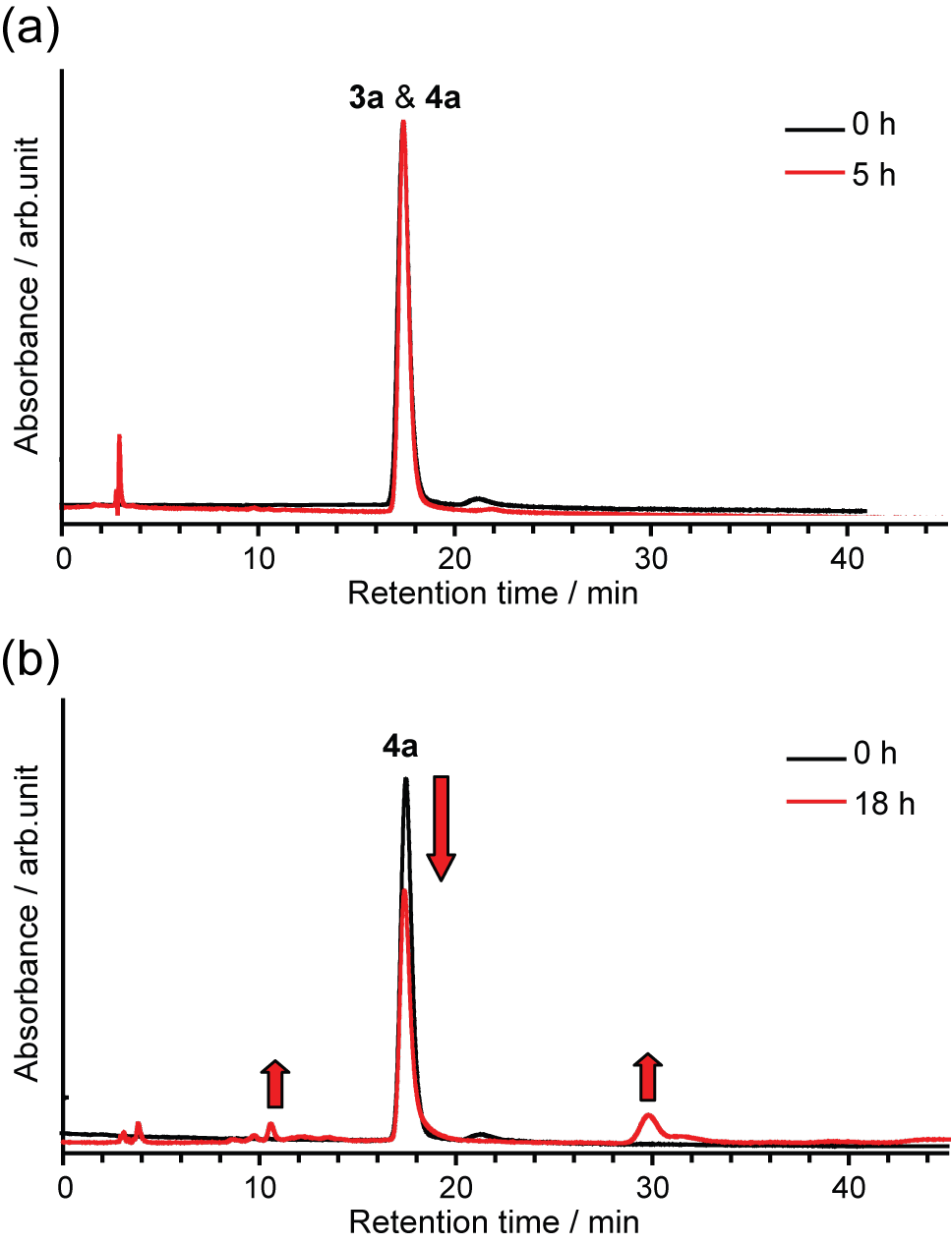
HPLC profiles of the mixture of **3a** and **4a**, (a) after heating in refluxing 1,2-dichlorobenzene and (b) after heating at 250 °C. Conditions: column, Buckyprep (Ø 4.6 mm × 250 mm); eluent, toluene; flow rate, 1.0 mL/min; wavelength, 330 nm; rt.

Further isolation of **4a** and **3b** was respectively accomplished using a combination of heating and HPLC separation. When the powdery mixture of **3a** and **4a** was heated to a temperature of 250 °C, selective decomposition of **3a** was observed (Figure 6). Since the peak of pristine La@C_82_ at ca. 30 min is observed after heating (see Figure 6b), most probably the detachment of the addend is taking place and a thermal isomerization thereafter. After HPLC purification of the crude reaction mixture, the number of the ^1^H NMR signals attributed to the methylene protons was reduced (Figure 5b). Furthermore, the ^1^H NMR spectrum of **4a** at 230 K unambiguously shows the existence of a single regioisomer, a [5,6]-adduct, which has *C_s_* symmetry showing one AB quartet of the one set of equivalent methylene protons. The existence of the [6,6]-adduct is excluded because the adduct must show at least two AB quartets based on its molecular symmetry.

**Figure 7:**
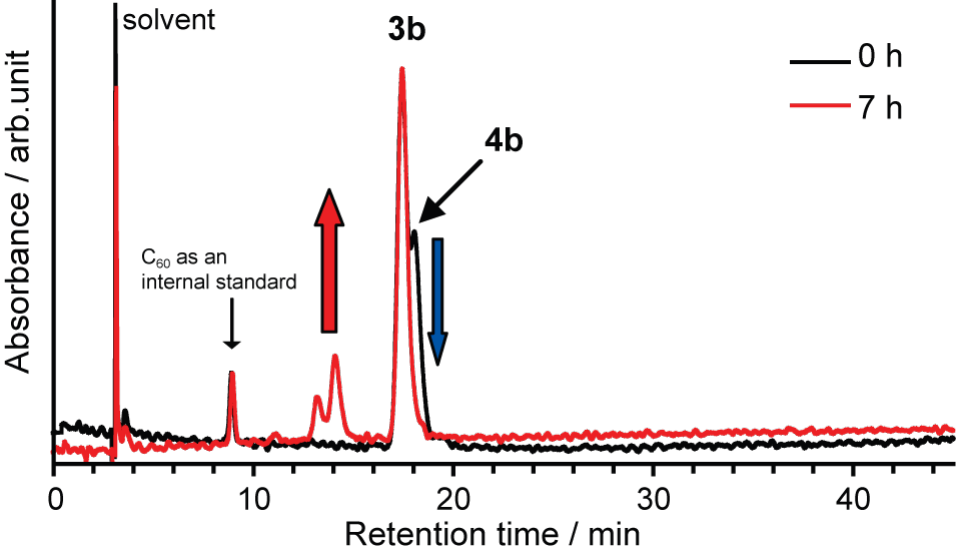
HPLC profiles of the reaction mixture of **3b** and **4b**, (black) before and (red) after heating in refluxing toluene. Conditions: column, Buckyprep (Ø 4.6 mm × 250 mm); eluent, toluene; flow rate, 1.0 mL/min; wavelength, 330 nm; rt.

In the case of purifying **3b**, selective decomposition of **4b** was observed at much lower temperature than that of **3a** and **4a**, after refluxing the mixture of **3b** and **4b** in toluene (Figure 7). This phenomenon is rationalized by the decomposition of the addend itself, because no pristine La@C_82_ was detected after the heating in contrast to the case of **3a** and **4a** (vide supra). Therefore, it is concluded that the addend of **4b** containing methoxy groups is thermally less stable than the addend of **3a** and **4a**. The following HPLC purification afforded isolated **3b**. This result suggests that **3b** is more stable against heating than **4b**.

**Figure 8:**
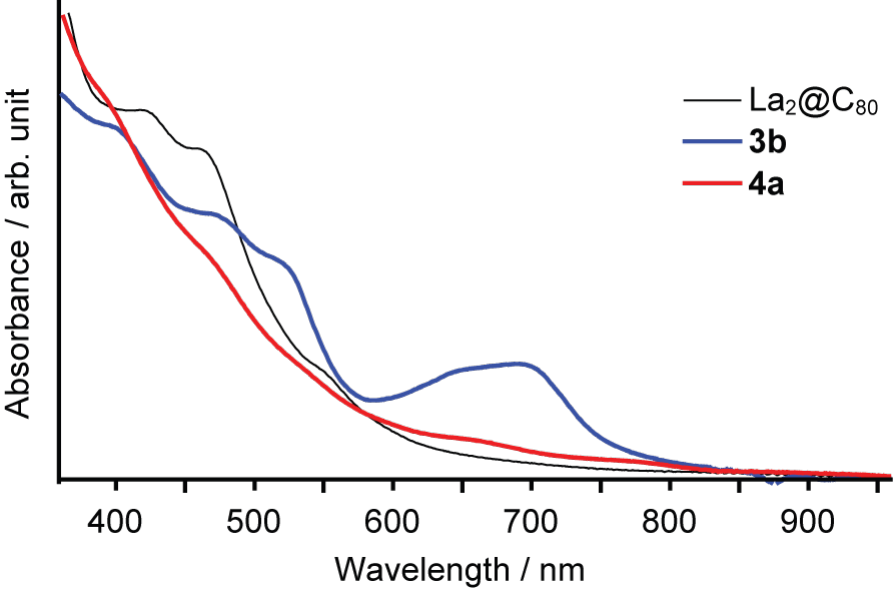
UV–vis/near-IR absorption spectra of **3b** and **4a** in toluene.

The UV–vis spectra of purified **4a** and **3b** were recorded using a spectrophotometer (UV-3150; Shimadzu Corp.) instead of the HPLC apparatus (Figure 8). The spectra reveal that **4a** shows no clear absorption peaks in the measuring range, and demonstrates a similar spectrum to those of [5,6]-adducts of La_2_@C_80_ [15]. This result shows good agreement with the ^1^H NMR spectrum, which indicates that **4a** is a [5,6]-adduct. However, **3b** shows a specific absorption band around 700 nm as in the spectrum recorded by the HPLC system shown in Figure 3, indicating its [6,6]-structure.

**Figure 9:**
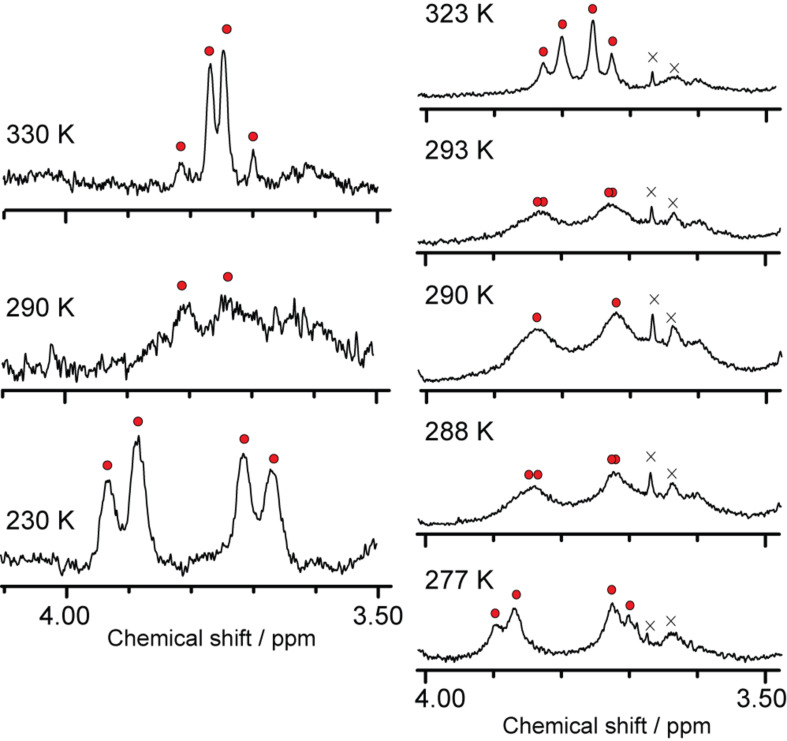
Temperature-dependent ^1^H NMR spectra of **4a** in C_2_D_2_Cl_4_ (left) at 300 MHz, and (right) at 500 MHz for precise analysis.

Temperature-dependent dynamics of the [4 + 2] adducts of endohedral metallofullerenes were studied for **4a** by dynamic ^1^H NMR measurements. Although **4a** did not show clear peaks at 290 K, distinct peaks were observed when the temperature was sufficiently lower or high enough distant from the coalescence temperature (*T*_c_) (Figure 9). This fact suggests that the boat-to-boat inversions of **4a** between the pentagon side and the hexagon side are sufficiently slow or fast to allow their observation in an NMR time scale. The signals from the AB-quartet of **4a** coalesce at 290 K (= *T*_c_) indicate a dynamic process, which is attributed to the boat-to-boat interconversion of the cyclohexane ring of the addend similarly to the related carbocyclic analogues of C_60_ adducts [12].

### Synthesis and characterization of La@C_82_ cycloadducts

**Scheme 2:**
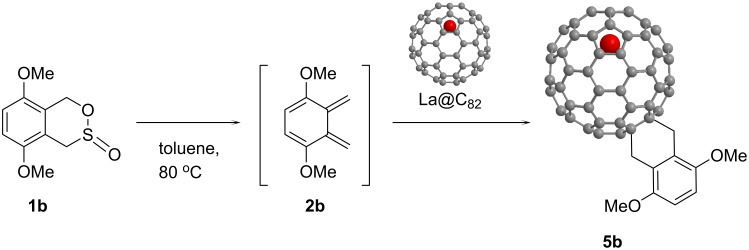
Synthesis of [4 + 2] adducts of La@C_82_.

**Figure 10:**
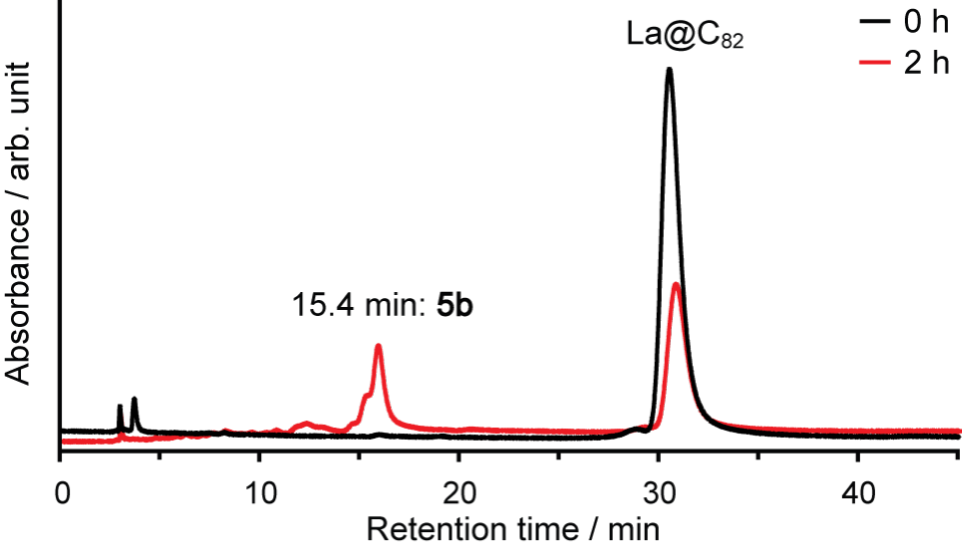
HPLC profiles of the reaction mixture for **5b**. Conditions: column, Buckyprep (Ø 4.6 mm × 250 mm); eluent, toluene; flow rate, 1.0 mL/min; wavelength: 330 nm; 40 °C.

**Figure 11:**
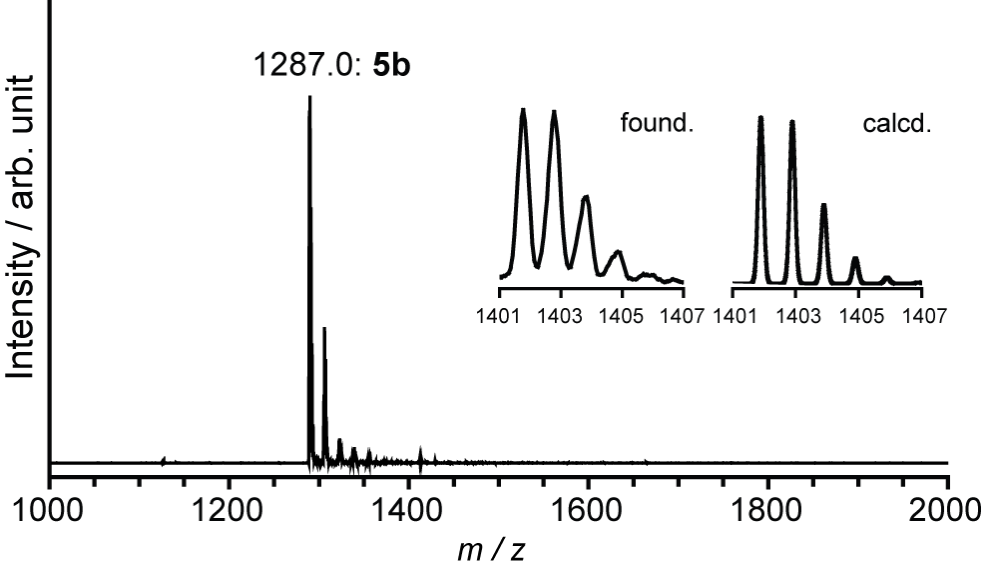
MALDI–TOF mass spectra (negative mode) of **5b**, using 1,1,4,4-tetraphenyl-1,3-butadiene as matrix.

Thermal reactions of **1b** and La@C_82_ afforded the [4 + 2] adduct **5b** (Scheme 2). **5b** was separated from the unreacted starting materials and byproducts through a one-step HPLC procedure (Figure 10). The MALDI–TOF mass spectrum of **5b** shows the peak attributed to the molecular ion peak (Figure 11).

**Figure 12:**
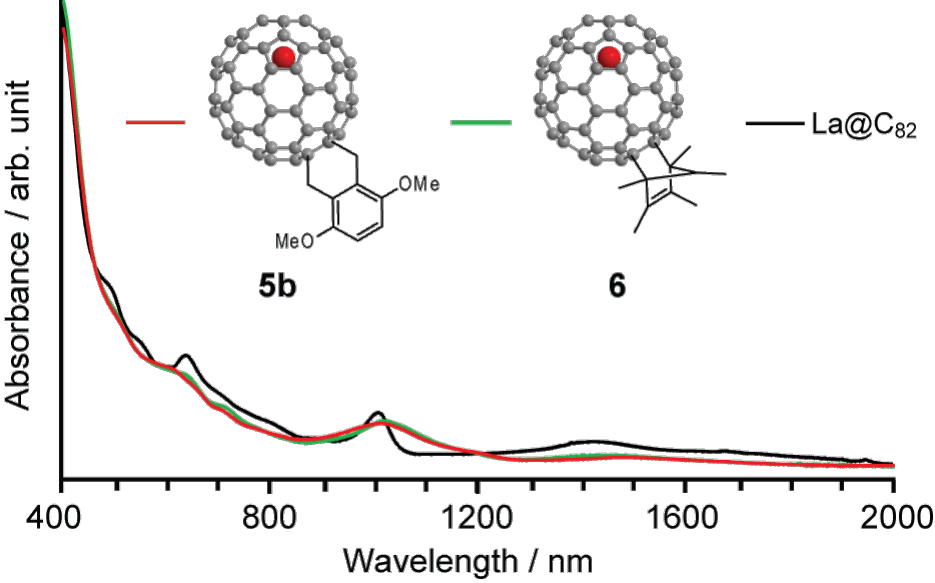
Vis–near-IR spectra of **5b**, **6** and La@C_82_ in CS_2_.

The electron spin resonance (ESR) spectrum of **5b** showed a unique octet signal (see Supporting Information File 1), indicating the paramagnetic property of **5b** as well as pristine La@C_82._ This result also indicates that the cycloaddition of the *o*-quinodimethane does not lead to a remarkable change in the electronic properties of the fullerene. This fact is supported by the vis–NIR absorption spectrum of **5b**, which retains the specific absorption bands of the pristine La@C_82_ (Figure 12). Broadening of the absorption bands is also observed, which is expected to be caused by the reduction of the molecular symmetry from *C*_2_*_v_* to *C*_1_ (vide infra).

**Figure 13:**
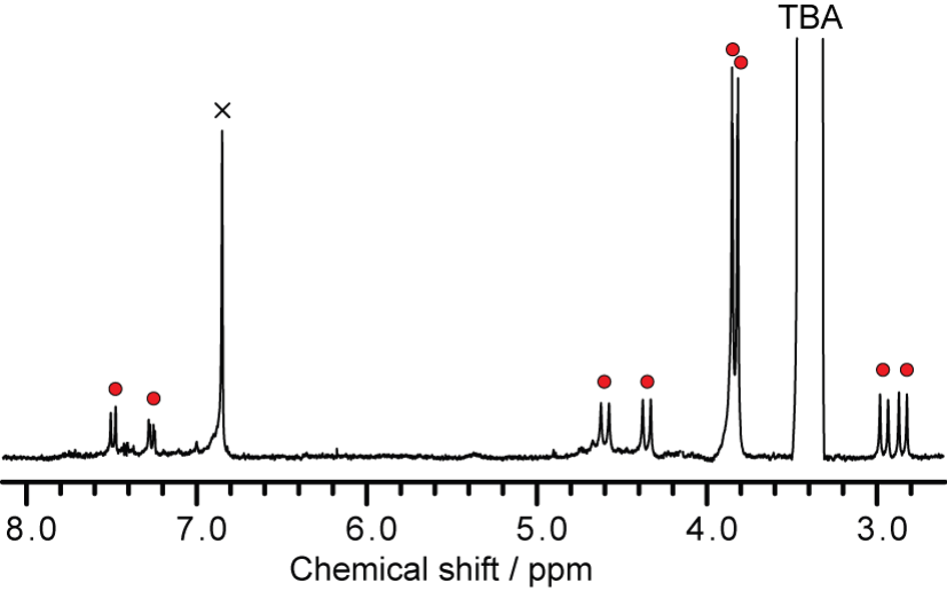
^1^H NMR spectrum of [**5b**]**^−^** in acetone-*d*_6_/CS_2_ (3/1 = v/v) at 223 K.

**Figure 14:**
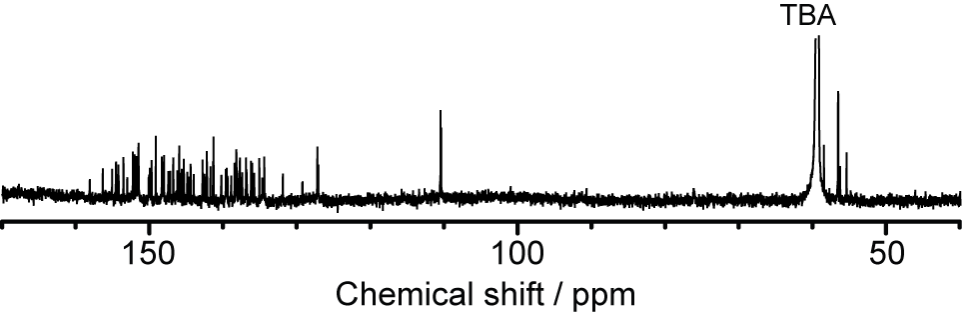
^13^C NMR spectrum of [**5b**]**^−^** in acetone-*d*_6_/CS_2_ (3/1 = v/v).

Further characterization of the molecular structure was performed using NMR measurements. Because **5b** has an open-shell electronic structure as well as pristine La@C_82_, **5b** was reduced electrochemically by one electron using bulk potential electrolysis for the NMR measurements. ^1^H NMR spectrum of the resulting anionic **5b** ([**5b**]**^−^**) clearly illustrates the characteristic signals from the addend (Figure 13). Signals of the methylene protons appear as a sharp AB system at 4.55, 4.30, 2.94, and 2.82 ppm. The ^13^C NMR spectrum demonstrates the total sum of 82 signals from the carbon cage (Figure 14), indicating *C*_1_ molecular symmetry for [**5b**]**^−^**. Signals at 58.2 and 56.0 ppm are attributed to the sp^3^ carbons of the addition position of the addend.

The vis–NIR absorption spectra provide information related to the molecular structure of **5b** (Figure 12). The resemblance between the spectra of **5b** and that of the previously reported [4 + 2] cycloadduct of La@C_82_ (**6**: La@C_82_Cp*) [11] in the vis–NIR region imply the isostructural characteristics of the respective compounds, which show the same addition pattern of the substituents. Although the structure of **5b** was not elucidated using X-ray crystallographic analysis, synthetic precedents with absorption spectral data and theoretical calculations [16] strongly suggest that the most feasible addition site of the addend is that indicated in Scheme 2.

**Figure 15:**
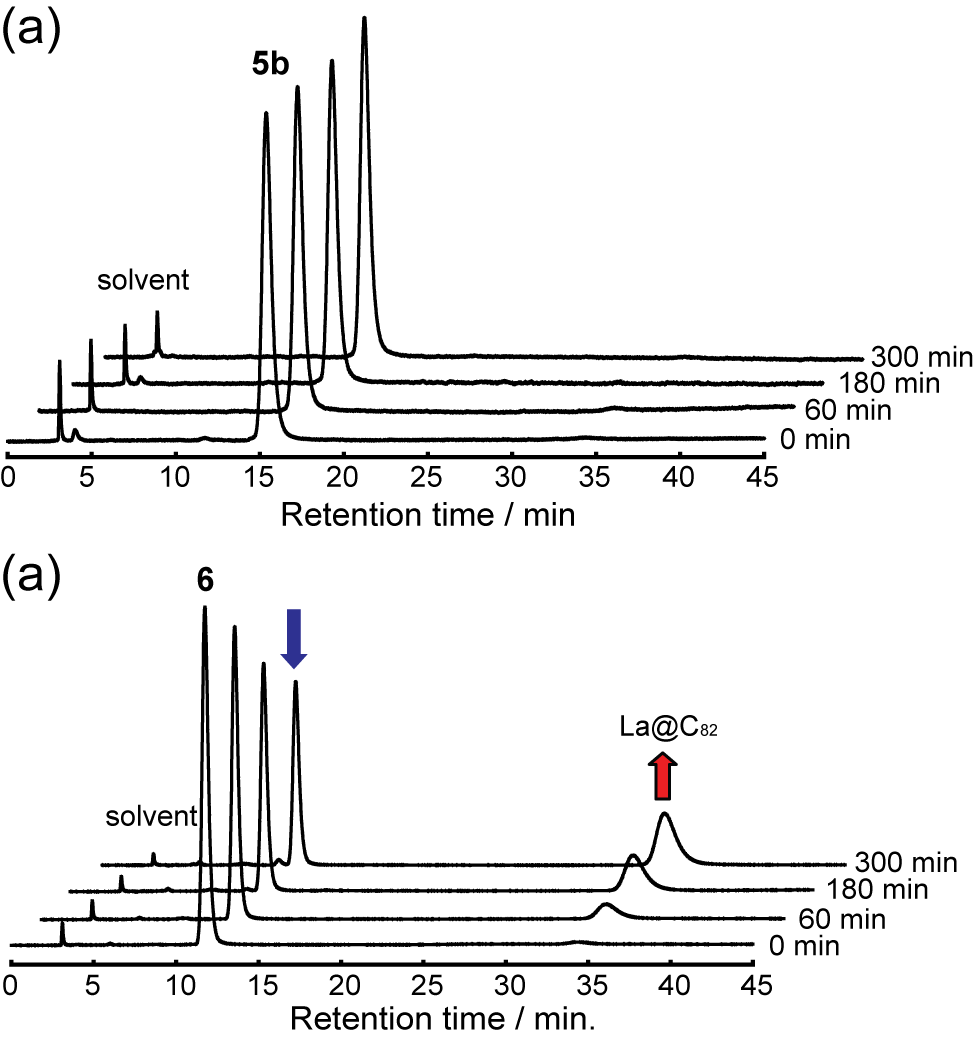
HPLC profiles for comparison of the thermal stabilities of (a) **5b** and (b) **6** at 30 °C. Conditions: column, Buckyprep (Ø 4.6 mm × 250 mm); temperature, 40 °C.

The thermodynamic stability of **5b** was evaluated by thermal heating. When a toluene solution of **5b** was let to stand at 30 °C, no decomposition was observed, whereas **6** showed decomposition and generation of pristine La@C_82_ (Figure 15). This result shows that using sultines, which generate reactive *o*-quinodimethanes and which afford cycloadducts, is an effective means to afford thermodynamically stable [4 + 2] adducts of La@C_82_.

## Conclusion

In summary, novel cycloadducts of La_2_@C_80_ and La@C_82_ were synthesized efficiently by a [4 + 2] cycloaddition reaction using sultines as a precursor of reactive *o*-quinodimethanes. Isolation of **3b** and **4a**, respectively, was achieved by the selective thermal decomposition of unstable isomers. The thermal stability of **5b** was also evaluated in comparison with **6**, and **5b** shows remarkable thermal stability. The molecular structures of the resulting compounds were characterized by spectroscopic analyses. Dynamic ^1^H NMR spectroscopic investigations of **4a** reveal temperature-dependent changes related to the conformational changes in the cyclohexane moiety generated upon reaction. The use of sultines for chemical modification of endohedral metallofullerenes has proved to be of general scope, being particularly useful to prepare thermally stable [4 + 2] cycloadducts.

## Supporting Information

Supporting information features detailed experimental procedures and spectral data for the compounds.

File 1 Descriptions on the synthesis and analyses of the compounds.
