# Supplementary material for: Thermodynamically stable [4 + 2] cycloadducts of lanthanum-encapsulated endohedral metallofullerenes
Source: Beilstein J Org Chem. 2014 Mar 25;10:714–21. doi: 10.3762/bjoc.10.65 (PMC3999875; doi:10.3762/bjoc.10.65)
Supplement: File 1 — Descriptions on the synthesis and analyses of the compounds. [file Beilstein_J_Org_Chem-10-714-s001.pdf]

Supporting Information

for

Thermodynamically stable [4 + 2] cycloadducts of lanthanum-encapsulated endohedral metallofullerenes

Yuta Takano[1], Yuki Nagashima[2], M. Ángeles Herranz[3], Nazario Martín[*3,4] and Takeshi Akasaka[*2,5,6]

Address: [1]Institute for Integrated Cell-Material Sciences (WPI-iCeMS), Kyoto University, Sakyo-ku, Kyoto 606-8501, Japan, [2]Life Science Center of Tsukuba Advanced Research Alliance, University of Tsukuba, Tsukuba, Ibaraki 305-8577, Japan, [3]Departamento de Química Orgánica I, Facultad de Química, Universidad Complutense, E-28040 Madrid, Spain, [4]IMDEA–Nanoscience, Campus de Cantoblanco, Madrid E-28049, Spain, [5]Foundation for Advancement of International Science, Tsukuba, Ibaraki 305-0821, Japan, [6]State Key Laboratory of Materials Processing and Die & Mould Technology, School of Materials Science and Technology, Huazhong University of Science and Technology, Wuhan 430074, China and Department of Chemistry, Tokyo Gakugei University, Tokyo 184-8501, Japan

Email: Nazario Martín - nazmar@quim.ucm.es; Takeshi Akasaka - akasaka@tara.tsukuba.ac.jp

* Corresponding author

Descriptions on the synthesis and analyses of the compounds

Table of Contents

Experimental section	S3
References	S7
Figure S1. HPLC profiles of isolated **3b**, **4a** and **5b**	S8
Figure S2. ^{13}C NMR spectra of **4a**	S8
Figure S3. HSQC spectra of **4a**	S9
Figure S4. ^{13}C NMR and DEPT 135 spectra of **5b**$^{-}$	S10
Figure S5. HMBC spectra of **5b**$^{-}$	S11
Figure S6. ESR spectra of **5b**$^{-}$	S12
Table S1. g-Value and hfcc of **5b**, **6** and La@C_{82}	S12

Experimental section

1.General

All solvents were dried according to standard procedures. Reagents were used as purchased, unless otherwise specified. High performance liquid chromatography (HPLC) was performed on an Agilent 1100 LC (Agilent Technologies) or an LC–9204 (Japan Analytical Industry Co., Ltd.). HPLC grade solvents were used as the eluents. Injection volumes of samples were 25 μL for analysis and 1500 μL for preparation. The ^{1}H and ^{13}C NMR measurements were carried out on a Bruker AVANCE 300 spectrometer, or a Bruker AVANCE 500 spectrometer with a CryoProbe system, where TMS was used as an internal reference (δ = 0.00 ppm). Absorption spectra were recorded in a SHIMADZU UV–3150 spectrophotometer or an Agilent 1100 Series diode-array detector. Matrix Assisted Laser Desorption Ionization (coupled to a Time–of–Flight analyzer) experiments (MALDI–TOF) were recorded on a Bruker REFLEX. The samples were prepared by drop casting of CS_2 solutions of the compounds and evaporation under ambient conditions. Electron paramagnetic resonance (EPR) spectra were recorded by a Bruker EMXPlus9.5/2.7. The spectrometer was operated at 9.8 GHz with a microwave power of 0.2 mW (30 dB), a modulation frequency of 100 kHz, and modulation amplitude of 300 mG. The *g* values were determined by the readout function of the spectrometer with the weak pitch as a standard, while the hyperfine splitting constants were measured using a Hall unit for calibration of the magnetic field.

Sultines **1**, **2**[S1], $La_2@C_{80}$[S2], $La@C_{82}$[S2], **6**[S3] were prepared according to the previously reported synthetic procedure and showed identical spectroscopic properties to those reported therein.

2.Synthesis of $La_2@C_{80}C_2H_4C_6H_4$ (3a and 4a)

A solution of $La_2@C_{80}$ (3.36 mg, 3.00×10^{-6} mol) and sultine **1a** (2.52 mg, 1.50×10^{-5} mol) in 75 mL of toluene was degassed by ultrasonication under reduced pressure, followed by stirring and heating at 80 °C under argon atmosphere. After 3 h, the reaction mixture was separated by one-step HPLC separation (a Buckyprep column, ϕ10 mm × 250 mm). The toluene used as an eluent of the HPLC process was removed under reduced pressure, then, the mixture of **3a** and **4a** was obtained as a mixture of [6,6] and [5,6] regioisomers of $La_2@C_{80}C_2H_4C_6H_4$ as a black solid.

The mixture of 3a and 4a: 73% yield (based on consumed starting fullerene from HPLC analysis). MALDI–TOF MS calcd. for $C_{88}H_6La_2$ ($[M]^-$): 1341.9, found: 1341.9 *m*/*z*.

2.1. Isolation of [5,6]-$La_2@C_{80}C_2H_4C_6H_4$ (4a) by heating

A powdered mixture of **3a** and **4a** (2.00 mg, 1.49×10^{-6} mol) was placed in a glass tube and sealed under vacuum. The tube was heated in an oil bath at 250 °C for 18 h, then, the tube was opened and the black solid in the tube was dissolved in CS_2. The solvent was replaced with toluene, and the solution was subjected to one-step HPLC to isolate **4a** from the resulting byproducts.

4a: 51 % yield. ^{1}H NMR (300 MHz, 2:1 (v/v) $CS_2/C_2D_2Cl_4$, 230 K) δ 7.64-7.43 (brs, 4H), 3.69 (AB quartet, δ_{AB} = 64.7 Hz, J_{AB} = 14.0 Hz, 4H); ^{13}C NMR (125 MHz, 3:1 (v/v) $CS_2/C_2D_2Cl_4$, 283 K) δ 154.8, 151.9, 148.5, 147.6, 146.9, 145.4, 145.0, 144.0, 143.7, 143.5, 143.1, 141.8, 141.7, 141.2, 140.5, 140.4, 140.2, 139.4, 139.1, 138.9, 138.8, 138.6, 138.4, 138.0, 137.9, 137.5, 137.4, 137.3, 137.2, 137.1, 136.2, 136.1, 136.0, 135.7, 135.7, 134.7, 134.7, 131.5, 130.5, 130.3, 128.7, 128.5, 128.0, 127.4, 127.0, 54.0

(sp^3 on cage), 46.9 (2C, CH_2).; MALDI–TOF MS calcd. for $C_{88}H_8La_2$ ($[M]^-$): 1341.9, found. 1341.9 *m*/*z*.

3.Synthesis of $La_2@C_{80}C_2H_4[C_6H_2(MeO)_2]$ (3b and 4b)

A solution of $La_2@C_{80}$ (1.68 mg, 1.50×10^{-6} mol) and sultine **1b** (1.71 mg, 7.50×10^{-5} mol) in 30 mL of toluene was degassed by an ultrasonicator under reduced pressure, followed by stirring and heating at 80 °C under argon atmosphere. After 2 h, the reaction mixture was separated by one-step HPLC separation (a Buckyprep column, ϕ 10 mm × 250 mm). The toluene used as an eluent of the HPLC process was removed under reduced pressure, then, the mixture of compound **3b** and **4b** was obtained in a purity of over 80%. Further purification of **3b** was achieved by heating the product in refluxing toluene, followed by an HPLC separation.

3b: 30% yield (based on consumed starting material from HPLC analysis). ^{1}H NMR (300 MHz, 2:1 (v/v) $CS_2/C_2D_2Cl_4$, 248 K) δ 6.87 (d, J = 8.2 Hz, 1H), 6.81 (d, J = 8.2 Hz, 1H), 3.88 (AB quartet, δ_{AB} = 298 Hz, J_{AB} 13.8, 2H), 3.47 (AB quartet, δ_{AB} = 184 Hz, J_{AB} 14.1, 2H); UV–vis/near-IR (toluene) λ_{max}: 689.0 nm; MALDI–TOF MS calcd. for $C_{90}H_{12}O_2La_2$ ($[M]^-$): 1401.9, found. 1401.8 *m*/*z*.

4b: 34% yield (based on consumed starting material from HPLC analysis). MALDI–TOF MS calcd. for $C_{90}H_{12}O_2La_2$ ($[M]^-$): 1401.9, found. 1401.9 *m*/*z*.

4.Synthesis of $La@C_{82}C_2H_4[C_6H_2(MeO)_2]$ (5b)

A solution of $La@C_{82}$ (5.00 mg, 4.50×10^{-6} mol) and 2 equivalents of sultine **1b** in 30 mL of toluene was degassed by a freeze-thaw process (three times) under reduced pressure, and then sealed. After stirring and heating at 80 °C under argon atmosphere

for 2 h, the reaction mixture was separated by one-step HPLC separation (a Buckyprep column, ϕ 10 mm × 250 mm). As the result, **5b** was obtained in 27% yield based on recovered La@C_{82}.

Preparation of the anionic 5b ([5b]$^-$) by bulk electrolysis.

[5b]$^-$ was prepared similarly as described in the literature.[S4]

Controlled-potential bulk electrolysis was performed using an H-type cell with two platinum gauze electrodes as the working and counter electrodes. [**5b**]$^-$ was obtained under argon in *o*-DCB containing 0.2 M $TBAClO_4$ by setting the applied potential at −800 mV, which is 300 mV more negative than the $E^{1/2}$ value of the [**5b**]$^-$/**5b** redox couple. The excess supporting electrolyte was precipitated from the solvent by adding carbon disulfide to the *o*-DCB and then removed by filtration. The solvent was evaporated under reduced pressure. The residual brown solid was washed with hexane and dried.

[5b]$^-$: ^{1}H NMR (300 MHz, acetone-d_6/CS_2=3/1, 223 K) δ 6.83 (s, 2H), 4.55 (d, J=14.4 Hz, 1H), 4.30 (d, J=14.1 Hz, 1H), 3.79 (s, 3H), 3.76 (s, 3H), 2.94 (d, 14.4 Hz, 1H), 2.82 (d, J=14.1 Hz, 1H); ^{13}C NMR (500 MHz, acetone-d_6/CS_2=3/1, 293 K) δ157.9, 156.1, 154.8, 154.3, 154.2, 154.0, 153.9, 153.3, 153.3, 152.8, 152.0 (2C), 151.8, 151.5 (2C), 151.3（2C）, 151.2, 149.9, 149.8, 149.7, 149.5, 148.9 (2C), 148.1, 147.8, 147.8, 147.2, 146.9, 146.5, 146.0, 145.7 (2C), 145.5, 145.4, 145.1, 144.6, 144.4, 144.2, 143.8, 142.5 (2C), 142.3, 142.0, 142.0, 141.9, 141.5, 141.1, 141.1 (2C), 140.0, 139.4, 139.4, 139.3, 139.2, 138.7, 138.2, 138.0, 138.0 (2C), 138.0, 137.8, 137.7, 137.5, 137.5, 137.4, 137.2, 136.7, 136.6, 136.6, 136.0, 135.8, 135.6, 135.5, 134.8, 134.5, 134.3, 134.2, 134.2,

131.7, 127.0, 127.0, 126.9, 126.8, 110.3,. 110.2, 58.2 (sp^3), 56.3 (CH_2), 56.3 (CH_2), 56.0 (sp^3), 36.4 (OCH_3), 34.4 (OCH_3); vis-near-IR (acetone/CS_2=3/1) λ_{max} 991, 919, 869, 610; MALDI–TOF MS *m*/*z*: Calcd for $LaC_{92}H_{12}O_2$ ($[M]^-$): 1286.99, Found: 1287.51.

References

S1. Illescas, B. M.; Martín, N.; Seoane, C. Ortí, E.; Viruela, P. M.; Viruela, R.; de la Hoz, A. *J. Org. Chem*. **1997**, *62*, 7585–7591.

S2. (a) Yamamoto, K.; Funasaka, H.; Takahashi, T.; Akasaka, T.; Suzuki, T.; Maruyama, Y. *J. Phys. Chem.* **1994**, *98*, 2008–2011. (b) Yamamoto, K.; Funasaka, H.; Takahashi, T.; Akasaka, T. *J. Phys. Chem.* **1994**, *98*, 12831–12833.

S3. Maeda, Y.; Sato, S.; Inada, K.; Nikawa, H.; Yamada, M.; Mizorogi, N.; Hasegawa, T.; Tsuchiya, T.; Akasaka, T.; Kato, T.; Slanina, Z,; Nagase, S. *Chem. Eur. J*. **2010**, *16*, 2193–2197.

S4. Takano, Y.; Aoyagi, M.; Yamada, M.; Nikawa, H.; Slanina, Z.; Mizorogi, N.; Ishitsuka, M. O.; Tsuchiya, T.; Maeda, Y.; Akasaka, T.; Kato, T.; Nagase, S. *J. Am. Chem. Soc.* **2009**, *131,* 9340.

(a)

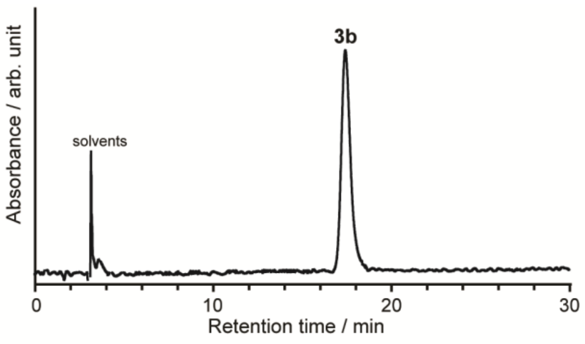

(b)

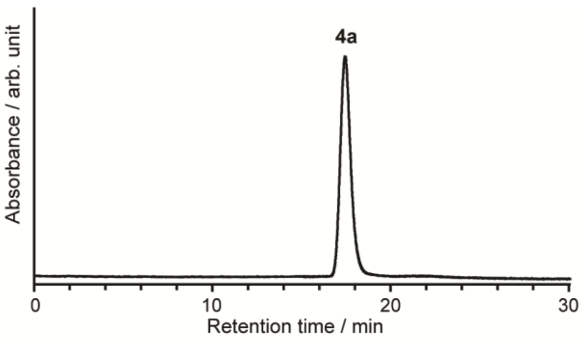

(c)

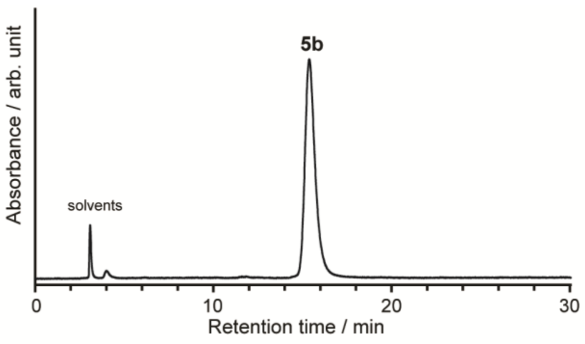

Figure S1: HPLC profiles of the isolated (a) **3b**, (b) **4a** and (c) **5b**. Conditions: column, Buckyprep (ϕ 4.6 mm × 250 mm); eluent, toluene; flow rate, 1.0 mL/min; wavelength: 330 nm; rt.

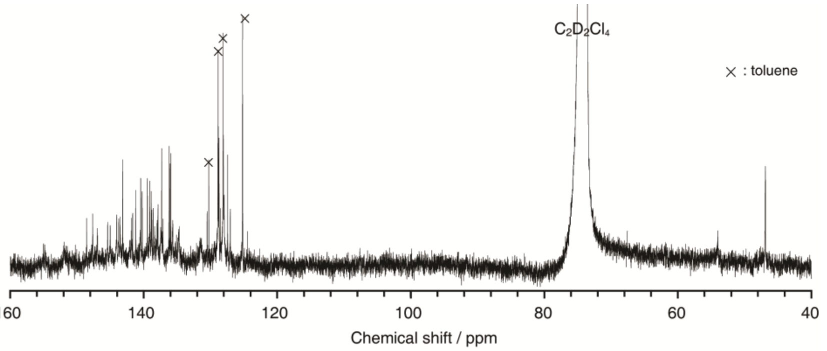

Figure S2: ^{13}C NMR spectra of **4a** in 2:1 (v/v) $CS_2/C_2D_2Cl_4$ at 283 K, at 125 MHz.

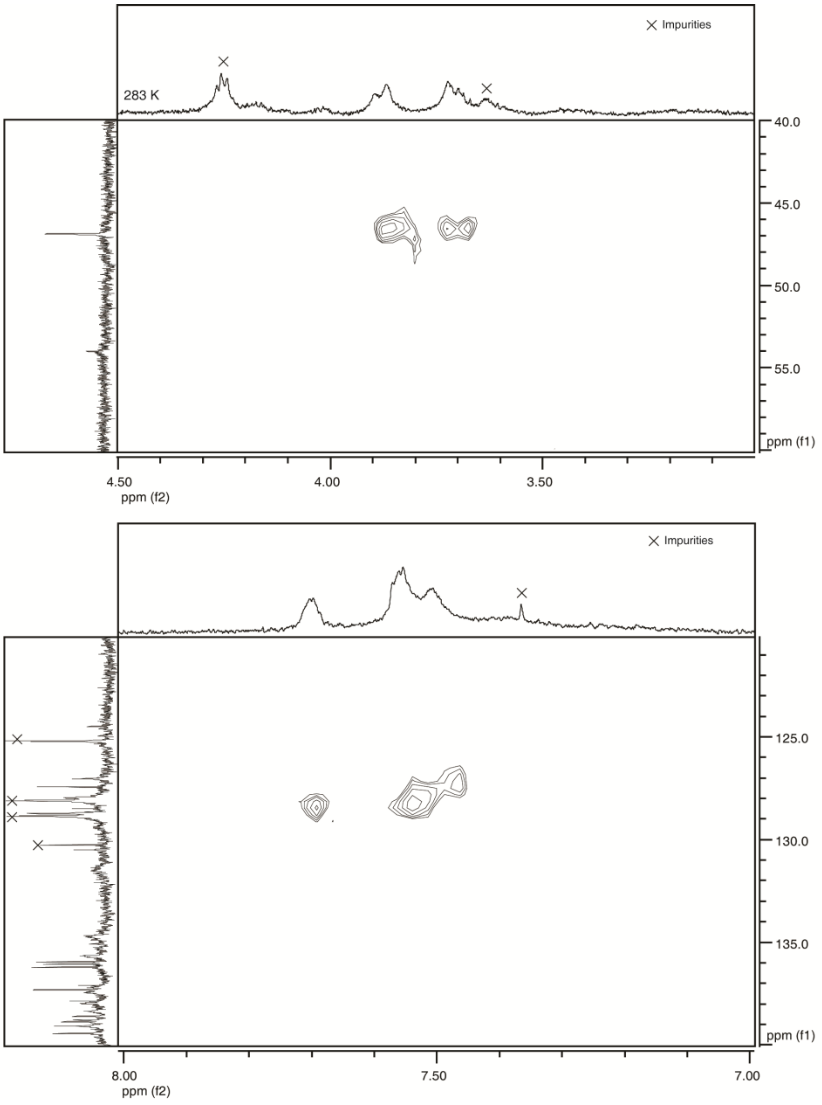

Figure S3: HSQC spectra of **4a** in 2:1 (v/v) $CS_2/C_2D_2Cl_4$ at 283K.

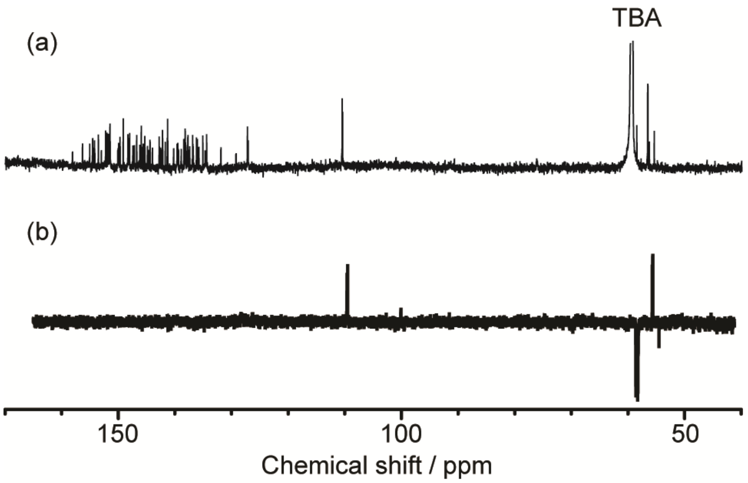

Figure S4: (a) ^{13}C NMR and (b) DEPT 135 spectra of *n*-Bu_4N^+[**5b**]$^-$ in acetone-d_6/CS_2(3/1) in 293 K.

(a)

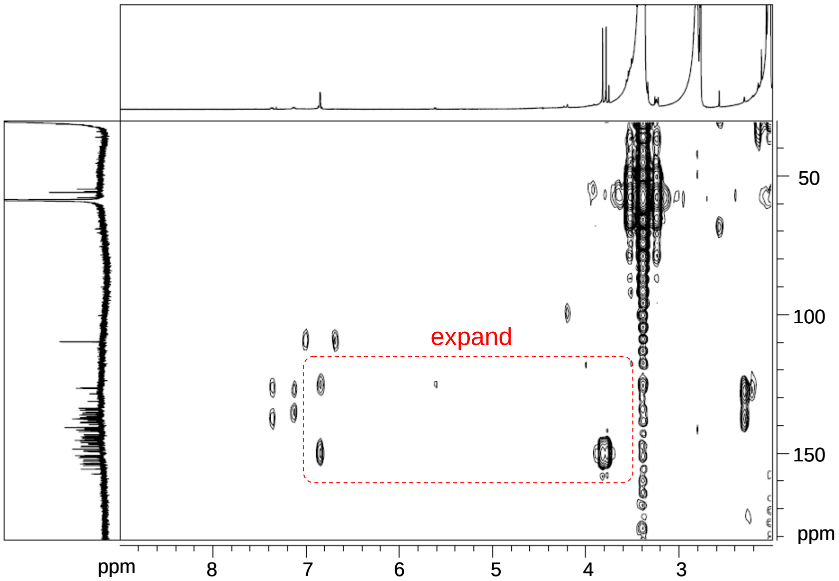

(b)

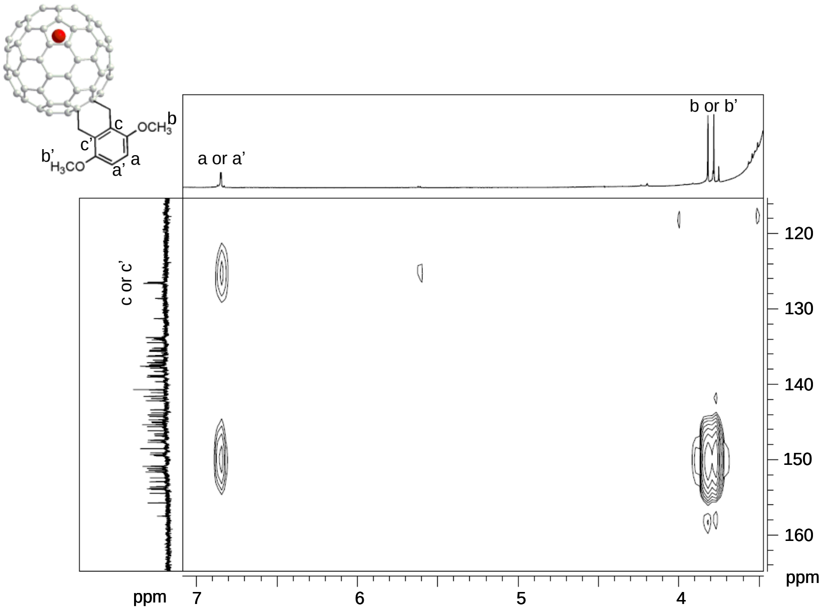

Figure S5: HMBC spectra of n-Bu$_4$N$^+$**5b**$^-$ in acetone-d_6/CS$_2$ (3/1) in 293 K. (a) Full region and (b) expanded spectra.

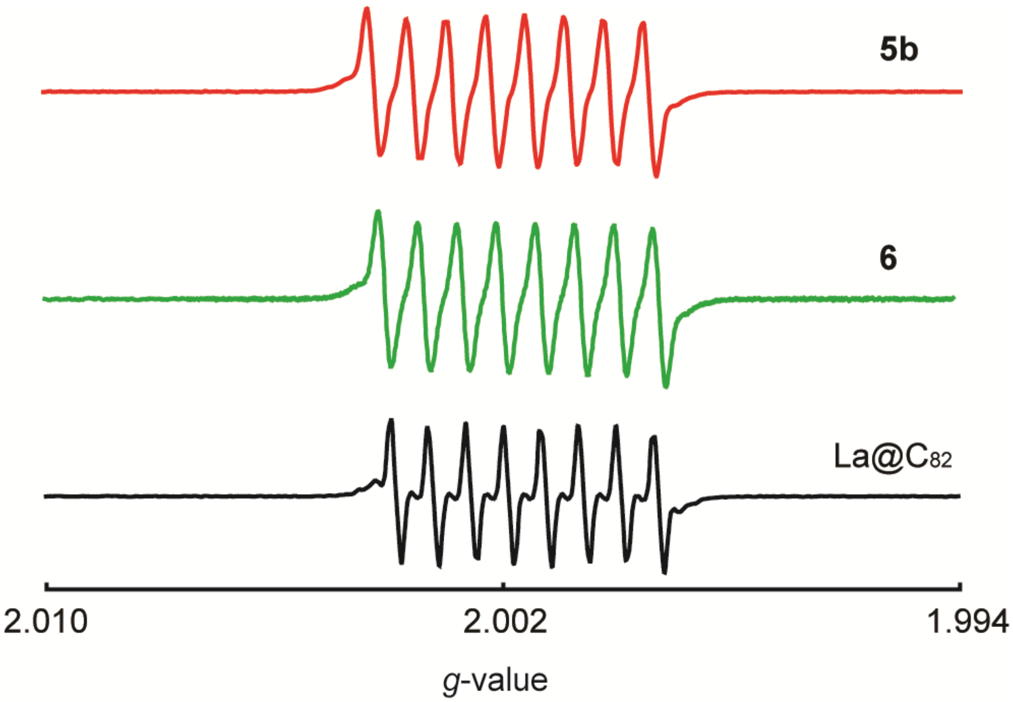

Figure S6. ESR spectra of **5b**, **6** and La@C_{82} in degassed toluene at rt.

Table S1: g-Value and hfcc of **5b**, **6** and La@C_{82}

	g-value	hfcc [G]
**5b**	2.0019	1.22
**6**	2.0018	1.18
La@C_{82}	2.0016	1.15
